# A novel traction device applying the “anchor traction method” during pharyngeal endoscopic submucosal dissection

**DOI:** 10.1055/a-2638-5469

**Published:** 2025-07-14

**Authors:** Keisaku Yamada, Masahiro Tajika, Tsutomu Tanaka, Nobuhito Ito, Akihiro Takagi, Yasumasa Niwa

**Affiliations:** 1 538357 Department of Endoscopy, Aichi Cancer Center, Nagoya, Japan

Endoscopic submucosal dissection (ESD) for pharyngeal cancer can be an effective method of preserving organ function. In this procedure, laryngeal forceps are used to directly grasp and pull the lesion; however, there are limitations in endoscopic manipulation owing to interference between the laryngeal forceps and the endoscope. Therefore, several methods that reduce interference have been reported 1 2 . We previously reported a new method using a multi-loop traction device (MLTD; Boston Scientific Co. Ltd., Tokyo, Japan), named the “anchor traction method” to enable multiple traction points in colorectal ESD 3 4 . Here, we report a useful technique in which this method was applied during ESD for pharyngeal cancer ( Video 1 ).

A novel traction device applying the anchor traction method during pharyngeal endoscopic submucosal dissection.Video 1

The lesion was a 15-mm 0–IIa lesion at the left pyriform sinus ( Fig. 1 ). After the otolaryngologist performed laryngeal expansion to create the space, marking and full-circumferential incision were done. The middle loop of the MLTD was attached to a reopenable clip (SureClip; MicroTech, Nanjing, China), and placed on the oral side of the lesion, and the two additional loops of the MLTD were then attached to the lesion, as previously reported in the anchor traction method ( Fig. 2 ). The otolaryngologist grasped the middle loop using laryngeal forceps, and effective traction could be applied ( Fig. 3 ). Successful traction was maintained with multiple points and en bloc resection was completed. Pathological analysis revealed that the lesion was squamous cell carcinoma pTis.

**Fig. 1:**
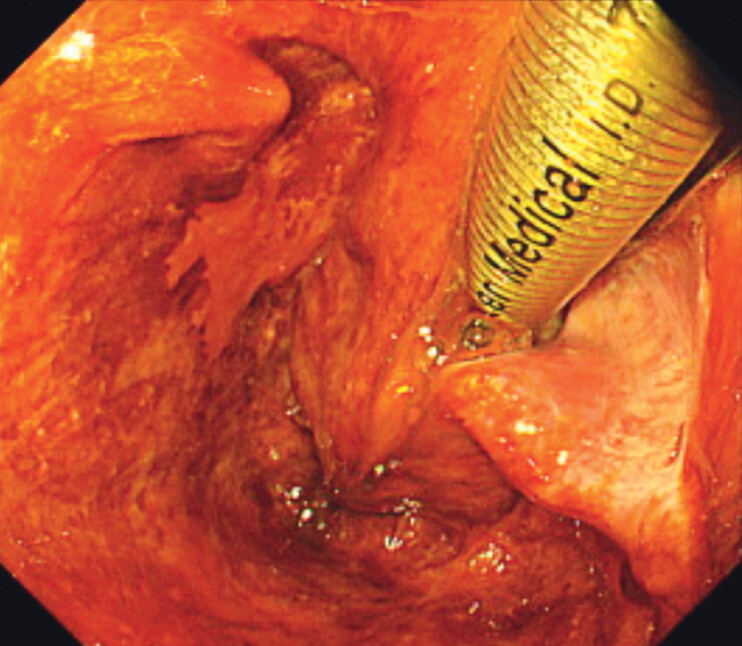
The lesion was a 15-mm 0–IIa lesion at the left pyriform sinus.

**Fig. 2:**
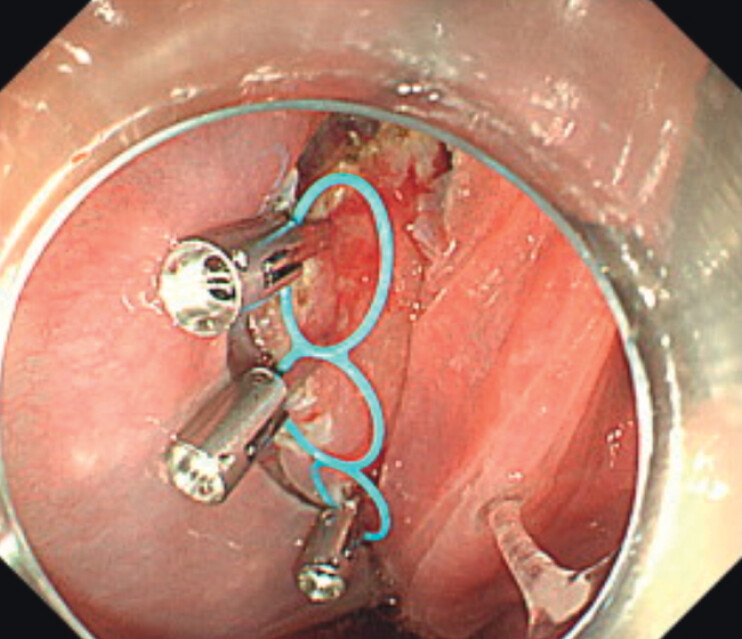
The multi-loop traction device was attached to the lesion as previously reported for the anchor traction method.

**Fig. 3:**
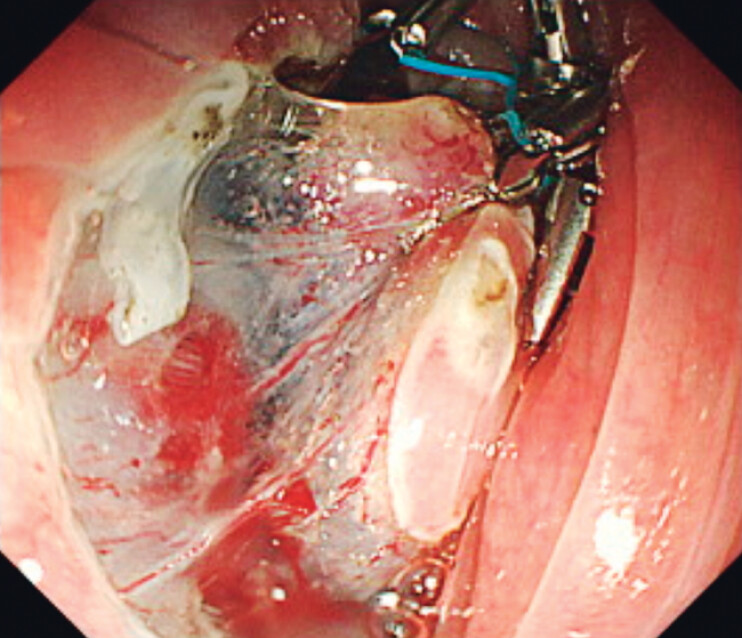
A good field of view was obtained with multi-point traction using the multi-loop traction device.

The advantages of this method are as follows. First, as with other methods 1 2 , the use of the MLTD reduces interference between the endoscope and forceps, and also decreases lesion damage by reducing the number of times the forceps must re-grasp the lesion. Furthermore, as the MLTD can be tractioned at three points, the field of view is better than with traction at a single point. This method is therefore potentially useful for ESD in pharyngeal cancer.

Endoscopy_UCTN_Code_TTT_1AO_2AG_3AD

## Notes

Endoscopy E-Videos https://eref.thieme.de/e-videos
*E-Videos* is an open access online section of the journal *Endoscopy* , reporting on interesting cases and new techniques in gastroenterological endoscopy. All papers include a high-quality video and are published with a Creative Commons CC-BY license. Endoscopy E-Videos qualify for HINARI discounts and waivers and eligibility is automatically checked during the submission process. We grant 100% waivers to articles whose corresponding authors are based in Group A countries and 50% waivers to those who are based in Group B countries as classified by Research4Life (see: https://www.research4life.org/access/eligibility/ ). This section has its own submission website at https://mc.manuscriptcentral.com/e-videos .
